# Are sex and gender dimensions accounted for in NICE guidelines? A systematic review of 223 clinical guidelines

**DOI:** 10.1136/bmjph-2024-002510

**Published:** 2025-07-17

**Authors:** Rachel Hulme, Owen Wilkinson, Ella Haythornthwaite, Veronia Benjamin, Hemali Matharu, Marina Politis, Kate Womersley, Robyn Norton, Alice Witt

**Affiliations:** 1 The George Institute for Global Health UK, London, UK; 2 Imperial College London, London, UK; 3 NHS Lothian, Edinburgh, UK; 4 The George Institute for Global Health, Sydney, New South Wales, Australia; 5 UNSW Sydney, Sydney, New South Wales, Australia

**Keywords:** Public Health, Sex Factors, Sociodemographic Factors, Health Personnel, Female

## Abstract

**Introduction:**

The importance of sex and gender in the diagnosis and management of health conditions is well established, but the extent to which sex and gender disaggregated evidence is integrated into UK clinical practice guidelines remains unknown. Recent reviews of clinical guidelines in Canada and Europe identified that limited and inconsistent attention was paid to sex and gender dimensions of health and illness. This study aimed to determine how the UK’s National Institute of Clinical Excellence (NICE) clinical guidelines account for sex and gender.

**Methods:**

The study reviewed all NICE guidelines categorised as ‘clinical guidelines’ by NICE (223), excluding those solely linked to single-sex conditions (26). Reviewers evaluated whether they included information on sex and/or gender dimensions of disease risk, presentation, investigations and management. They also examined if sex and/or gender dimensions were considered outside of pregnancy, and how the gender of guideline committee chairs and members corresponded to how well sex and/or gender were accounted for.

**Results:**

Of 197 guidelines reviewed, 120 (61%) referenced sex and/or gender dimensions, with 81 (41%) referencing these dimensions outside of pregnancy and childbearing. A minority of guidelines mentioned sex and/or gender dimensions related to disease pathophysiology (2%), clinical presentation (9%), investigations (15%) and epidemiology (19%). 162 guidelines published details of their committee chairs, and 126 (76%) were men. Committees chaired by women tended to produce guidelines, which scored better for consideration of sex and gender.

**Conclusion:**

This study highlights key gaps in NICE guidelines which must be addressed through systematic, whole-sector progress to integrate sex and gender disaggregated research into clinical guidelines. As the single focal point responsible for guideline development in England and Wales, NICE has a unique opportunity to establish robust mechanisms to routinely embed this important evidence in guidelines. Multiple initiatives are recommended to identify relevant existing evidence across all clinical specialties.

**WHAT IS ALREADY KNOWN ON THIS TOPIC**

**WHAT THIS STUDY ADDS**

- This study identified key gaps in how the UK’s National Institute of Clinical Excellence (NICE) clinical guidelines account for sex and gender across all clinical specialties, and all aspects of disease presentation and management.
- Outside of pregnancy and childbearing, less than half of guidelines (41%) made any reference to sex or gender dimensions of disease. This study is the first to review NICE guidelines from this perspective.

**HOW THIS STUDY MIGHT AFFECT RESEARCH, PRACTICE OR POLICY**

- This study calls on NICE to invest in addressing these critical gaps, including leading initiatives to identify and embed relevant existing evidence on sex and/or gender dimensions across existing and future guidelines. Initiatives should include an extensive consultation, systematic review and engagement with leading experts to identify the relevant existing evidence. NICE should also publish recommendations to highlight the most urgent data gaps and establish robust mechanisms to embed key sex and gender disaggregated evidence into future guidelines.

## Introduction

 Sex and gender play fundamental roles in shaping individual and population health. Across a spectrum of medical conditions, from dementia and cardiovascular disease to neurodivergence and autoimmunity, sex and gender impact disease risk, presentation and progression, as well as treatment efficacy, side effects and dosing.^1^

Historically and today, limited attention has been paid to sex and gender dimensions in medical research, leading to inequities in clinical care and health outcomes.^2^ For example, women with acute myocardial infarction are 50% more likely to be misdiagnosed than men,^3^ experiencing higher early mortality and reduced long-term survival.^4^ Men experience substantially higher mortality than women after a hip fracture, where cumulative mortality at 12 months compared with the general population is 37.1% higher in men and 26.4% higher in women.^5^ 41% of transgender people report that healthcare staff lack understanding about their health needs, particularly regarding how gender-affirming hormone therapies may interact with other medical conditions or treatments.^6^ Similar gaps exist for patients who are intersex or have variations of sex characteristics.^7^

Clinical guidelines synthesise evidence across a range of medical specialties to set the standard for clinical best practice and to promote uniformly high-quality care.^8^ Guidelines aim to remove subjectivity and associated room for bias and assumptions in favour of evidence-based care. In the UK since 1999, guidelines are developed by expert committees and hosted by the National Institute for Health and Care Excellence (NICE), an executive non-departmental public body that is funded by and accountable to the UK Government’s Department of Health and Social Care.

Recent reviews of clinical practice guidelines in Canada^9^ and Europe^10^ identified that limited attention is paid to sex and gender dimensions. There are also inconsistencies in how sex and gender dimensions are integrated across these guidelines. Canadian and European government funders have led the way in promoting a sex and gender lens in health and illness through adoption of policies for research design in 2011 and 2016, respectively.^11^ In the UK, the Government’s Medical Research Council introduced the first sex and gender policy in 2023.^12^ Since 2023, over 16 funders, including the government’s National Institute for Health and Care Research, are collaborating through the Medical Science Sex and Gender Equity (MESSAGE) project to improve practice through sector-wide design and implementation of policies.^13^

This study sought to understand how sex and gender considerations are currently integrated into NICE guidelines, which steer UK clinical practice.

## Materials and methods

### Outcomes

The primary outcome of the review was the number (and proportion) of NICE clinical guidelines that referenced sex and/or gender dimensions and the number (and proportion) that referred to sex and/or gender dimensions outside of pregnancy and childbirth. Secondary outcomes included whether the terms ‘sex’ and ‘gender’ were used accurately; whether reference was made to trans, non-binary or intersex individuals and whether sex and/or gender considerations were included in reference to disease epidemiology and risk factors, pathophysiology, clinical presentation, investigation and/or management. Additionally, we reviewed the gender composition of the expert committee chairs and members who designed the guidelines across NICE’s ‘Conditions and Diseases’ and ‘Health and Social Care’ groupings and determined how these variables correlated with consideration of sex and gender dimensions.

### Data sources and inclusion criteria

**Figure 1:**
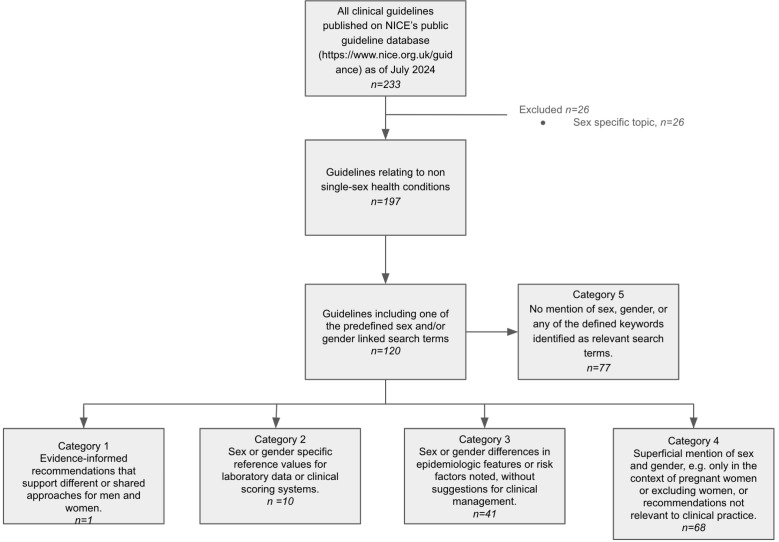
PRISMA flowchart recording data sources and inclusion criteria. PRISMA, Preferred Reporting Items for Systematic Reviews and Meta-Analyses

This review included all 223 clinical guidelines published on NICE’s publicly accessible guideline database (https://www.nice.org.uk/guidance) as of July 2024. Clinical guidelines were identified via the database by using database filters. We selected the type as ‘Guidance’ and the Guidance programme as ‘Clinical guidelines’. Clinical guidelines for single-sex health conditions, such as twin and triplet pregnancy, menopause and prostate cancer, were excluded (figure 1). Exclusion of single-sex health conditions was determined through consensus by two independent reviewers. NICE clinical guidelines extracted in this way will be referred to as ‘guidelines’ in this article. We use ‘sex’ to denote biological characteristics and ‘gender’ to reflect the roles, behaviours and identity a person assumes in society, affecting the power, privileges and constraints they might experience.^14^ Throughout we refer to people’s gender using the terms ‘women/men/non-binary/trans’ and their sex with the terms ‘female/male/intersex’. When citing the work of others, including NICE clinical guidelines, we have used the same sex- and/or gender-linked terminology deployed by the referenced source.

### Data extraction

We designed a framework to assess if sex and/or gender dimensions had been accounted for, informed by similar studies conducted in Canada,^9 15 16^ Australia^17^ and across Europe.^10^ All reviewers used a standardised form (see online supplemental materials) to extract the title, NICE reference code, publication date and most recent update date and committee details from each NICE guideline.

Reviewers searched guideline text electronically for the keywords “sex,” “gender,” “male,” “female,” “men,” “women,” “man,” “woman,” “boy,” “girl” and “pregnan*,” “Menopause,” “fertility,” “breast,” “ova,” “vulva,” “vagina,” “cervi*,” “uter*,” “penis,” “testic*,” “teste*,” “prostat*,” “oestrogen,” “testosterone” and “progest*.”

Reviewers extracted the name, role and presumed gender of the most recent committee members. The committee roles were chair, committee member and lay member. We identified presumed gender (categories: man, woman and other) based on the latest committee membership documents published by NICE and gendered titles (eg, Miss, Mr, Mrs and Ms). When the guideline did not provide this information, we used the open-source online tool gender API (https://www.genderapi.io/) to provide a statistical probability as to which gender a given first name was most likely associated with.

### Data synthesis and analysis

Four reviewers (OW, VB, HM and EH) undertook a preliminary analysis of 37 guidelines. An initial sample was selected according to clinical subjects which the group agreed were priority areas for consideration. Reviewers then convened to resolve inconsistencies in data extraction by consensus and refine the framework. Reviewers rated each guideline using a standardised category system (table 1), where ‘Category 1’ represented the most consideration of sex and gender (evidence-informed recommendations that support different or singular approaches for men and women) and ‘Category 5’ represented the least consideration of sex and gender (no mention of sex, gender or any of the defined keywords identified as relevant search terms). Refinements included expansion of the range of keywords and of the ‘Category 3’ grading criteria to include sex and/or gender disaggregated risk factors alongside references to epidemiological differences. The ‘Category 2’ grading criteria were also expanded to include sex and/or gender disaggregated clinical scoring systems, alongside sex differentiated laboratory values.

**Table 1:** Table recording the defined criteria for each rating category

Category number	Guideline content
**1**	Evidence-informed recommendations that support different or singular approaches for men and women.
**2**	Sex- or gender-specific reference values for laboratory data or clinical scoring systems.
**3**	Sex or gender differences in epidemiological features or risk factors without suggestions for clinical management.
**4**	Superficial mention of ‘sex’ and/or ‘gender’, for example, only in context of pregnant women or excluding women, or recommendations not relevant to clinical practice.
**5**	No mention of sex, gender or any of the defined keywords identified as relevant search terms.

Five reviewers (OW, VB, HM, EH and RH) analysed the data extracted through the keyword search, assessing the type, amount and applicability of evidence presented on sex and gender dimensions of epidemiology, risk factors, pathophysiology, clinical features, diagnosis and investigations and treatment and management. Reviewers rated each guideline using a standardised category system (table 1).

Two reviewers (combinations of OW, VB, HM, EH and RH) independently assessed for accuracy of sex- and/or gender-linked terminology, and binary use of sex and gender terminology across all guidelines, which considered sex and/or gender beyond pregnancy or childbirth. A second reviewer also checked the rating category for all guidelines achieving a ‘Category 1 or 2’ rating, as these were deemed to be the most dependent on reviewer judgement. Inter-reviewer variability was resolved by consensus and any conflicts were resolved by an independent third reviewer (AW). All other variables, including committee composition, and disease aspect considered were single reviewed. Reviewers were encouraged to raise any queries for resolution by RH and AW. Reviewers were also encouraged to read the text surrounding any terminology identified, record associated language and analyse relevant content to draw out key qualitative themes and trends.

Findings were analysed using Microsoft Excel pivot tables to explore general trends in guideline category ratings across time, and the gender of committee chairs, members and lay representatives. NICE organises clinical guidelines into ‘Conditions and Diseases’ (174) or ‘Health and Social Care Delivery’ (23). Conditions and Diseases groupings broadly correlate to medical specialties and body systems, such as cardiovascular, respiratory or liver conditions. ‘Health and Social Care Delivery’ categories include categories, such as children’s social care, mental health services and safeguarding

## Results

Of a total 223 NICE clinical guidelines, 197 covered non sex-specific conditions and were included in this review. Of these, 120 guidelines (61%) included information on sex and/or gender dimensions and 81 (41%) referenced sex and/or gender dimensions outside pregnancy and childbirth considerations.

**Figure 2:**
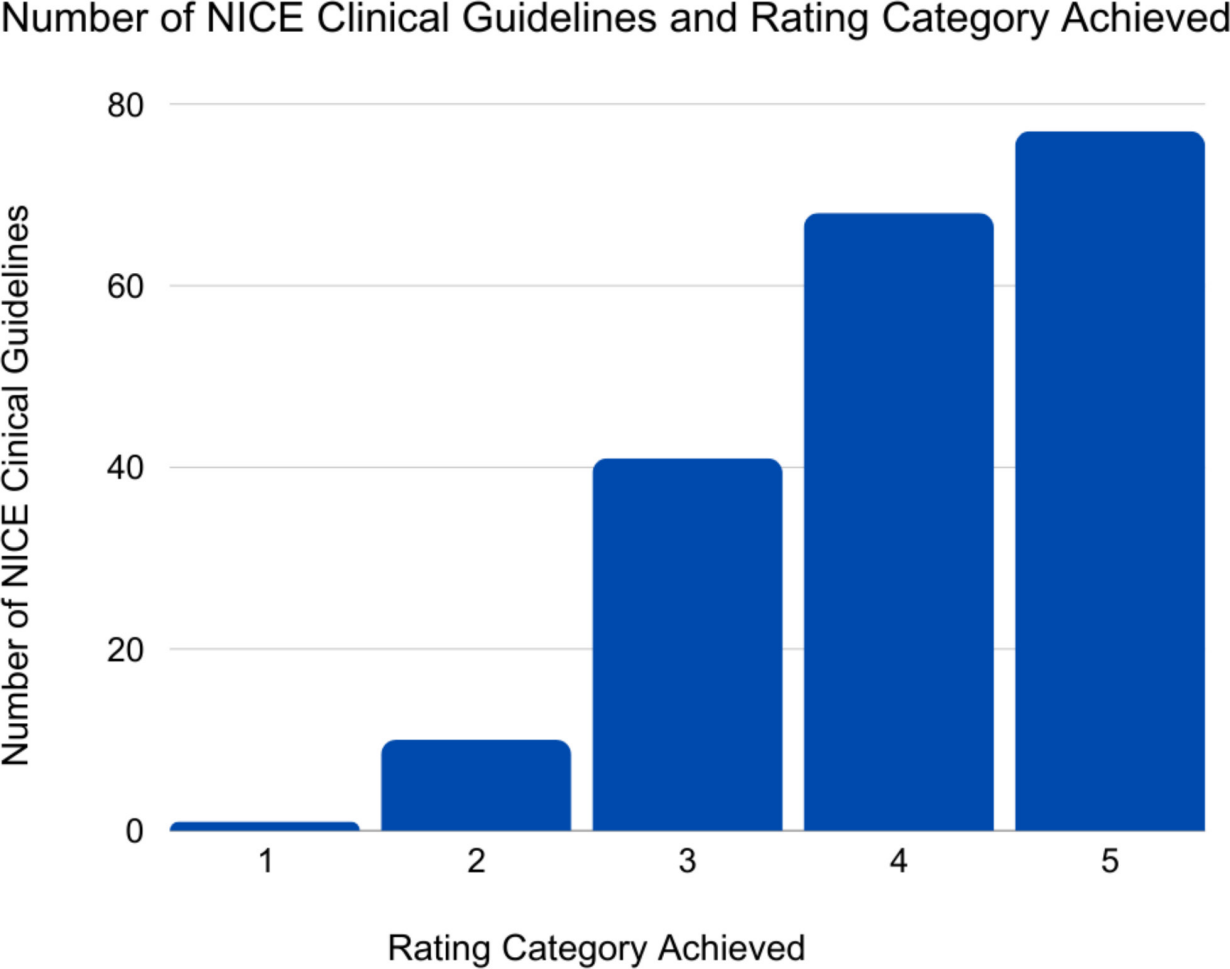
Number of NICE clinical guidelines according to the rating category which they achieved. NICE, National Institute of Clinical Excellence.

Of the 197 reviewed guidelines, only 1 guideline, a guideline addressing fertility issues (CG156), achieved a ‘Category 1’ rating (0.5%), meaning it provided evidence-informed recommendations supporting different or shared approaches for men and women (figure 2). Ten guidelines (5%) included sex and/or gender-specific reference values for laboratory data or clinical scoring systems (‘Category 2’). 77 guidelines (39%) made no mention of sex or gender dimensions (‘Category 5’) and 68 (35%) made only superficial mention of sex and/or gender dimensions, for instance only in the context of pregnant women (‘Category 4’). The average rating across reviewed guidelines was 4.07, where 5 represented the least consideration of sex and gender dimensions, and 1 represented the most (table 1).

**Figure 3:**
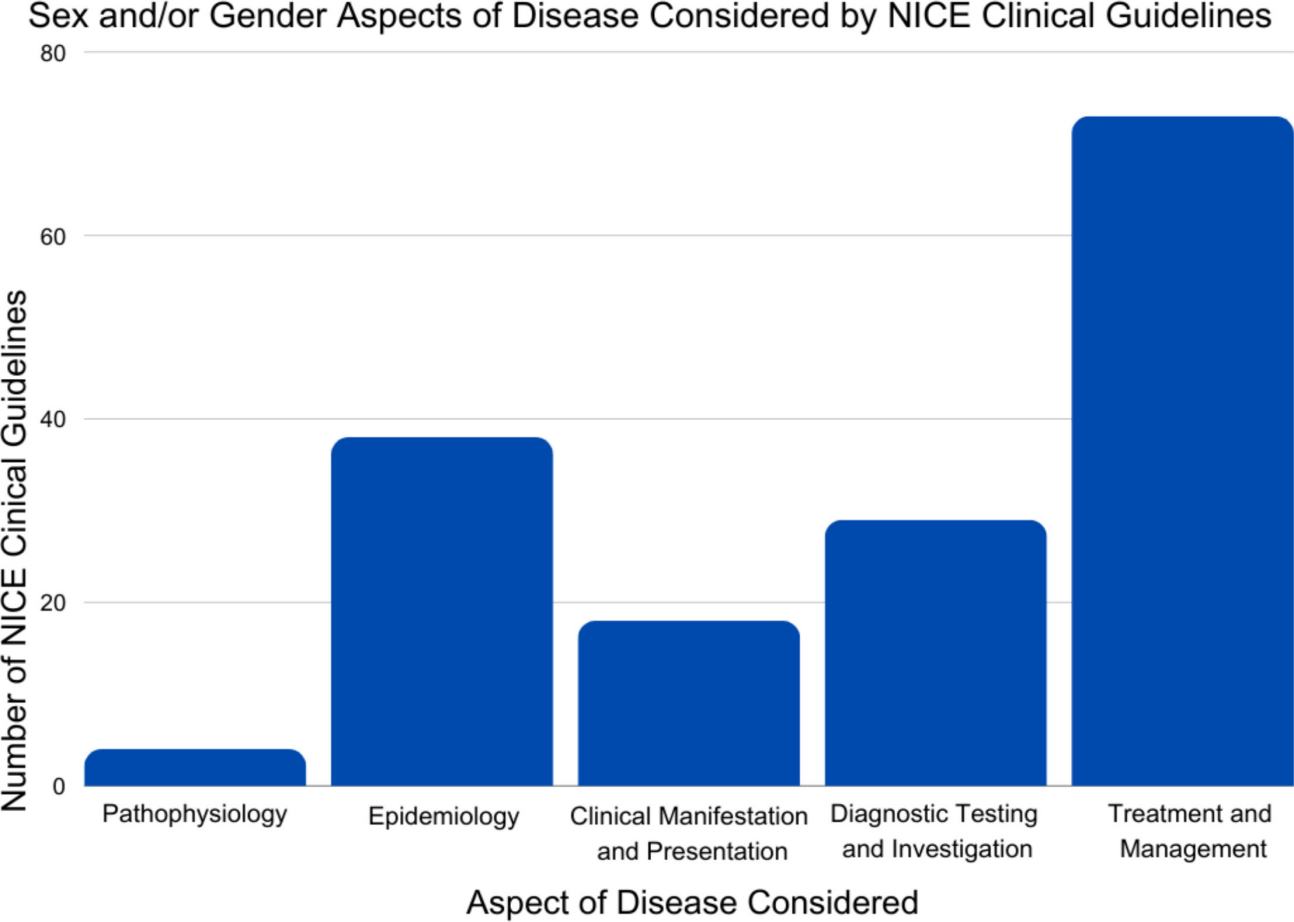
Number of NICE clinical guidelines considering the sex and/or gender dimensions of different disease components. NICE, National Institute of Clinical Excellence.

Only four guidelines (2%) referenced sex and/or gender dimensions of disease pathophysiology (figure 3). Examples include the role of menopause in worsening bone density in women (CG146) or the explanation that ‘it is likely that a significant proportion of male infertility results from abnormalities of genes on the Y chromosome involved in the regulation of spermatogenesis, and couples should be informed of this’ (CG156).

### Different ways of addressing disease

18 guidelines (9%) mentioned sex and/or gender dimensions of clinical manifestations and presentation of disease. The cystic fibrosis guideline (NG78) cited differential disease presentations relating to reproduction, stating that ‘people with cystic fibrosis are at risk of the following common complications: male infertility caused by obstructive azoospermia (almost all males with cystic fibrosis are infertile) and reduced female fertility’. Notably, two cardiovascular guidelines (CG95 and CG126) emphasised that the ‘symptoms of acute coronary syndromes of stable angina should not be assessed or treated differently across men and women’.

29 guidelines (15%) referenced sex- and/or gender-specific considerations for diagnostic testing and investigations. Such guidelines frequently included clinical scoring systems with different threshold values for men and women, respectively. For instance, in frailty assessment, addressed in the multimorbidity guideline (NG56), women required higher scores, according to the physical activity scale for the elderly. In the guidelines for physical health of people in prison (NG57) and mental health of adults in contact with the criminal justice system (NG66), women required a lower score to be diagnosed with mental health problems, according to the Correctional Mental Health Screen for Women (CMHS-W) or Correctional Mental Health Screen for Men (CMHS-M) tests, respectively. Women also required a lower alanine aminotransferase (ALT) derangement to qualify for liver biopsy according to the guideline for chronic hepatitis B (CG165). In the breast cancer guideline (NG101), a condition which predominantly (99%) impacts women,^18^ the guideline explicitly stated that the PREDICT tool, which is used to estimate prognosis and the absolute benefits of adjuvant therapy, is ‘not validated in men’. In this way, guidelines clarified when evidence relating to men was unavailable. Reviewers did not identify any comparable instances where guidelines clarified when evidence was missing for female patients.

38 guidelines (19%) included some reference to sex and/or gender differences in disease epidemiology or risk factors. Guidelines featured epidemiological examples of conditions, which are more prevalent in women and/or females (such as breast cancer (NG101), myalgic encephalomyelitis (or encephalopathy)/chronic fatigue syndrome (ME/CFS) (NG206) and depression (NG222)) as well as conditions more prevalent in male patients (such as newborn cardiopulmonary dysplasia (NG124), bladder cancer (NG2) and substance abuse in depression (NG222)).

73 guidelines (37%) mentioned sex or gender dimensions in relation to treatment and management. 42 of these (58%), equating to 21% of the total 197 guidelines reviewed, related solely to management in pregnant and breastfeeding women and typically specified drugs or procedures to be avoided. Drug-linked recommendations were common, some of which were included within the guideline itself, such as avoiding angiotensin-converting enzyme inhibitors for pregnant patients with hypertension (NG136). Other recommendations regarding medicines referenced supplementary materials, such as the British National Formulary or updated Medicines and Healthcare Products Regulatory Agency guidance on antiepileptic drugs in pregnancy. Sex and gender dimensions to consider in treatment and management regarding males were similarly focused on reproduction, such as the hyperthyroidism guideline, which advises against radioactive iodine treatment for adults with Grave’s disease ‘trying to … father a child within the next 4 to 6 months’.

### NICE groupings

Guidelines grouped by NICE as ‘Conditions and Diseases’ achieved a better average rating for sex and/or gender considerations (3.99) than the guidelines within the ‘Health and Social Care Delivery’ grouping (4.65).

We analysed ‘Condition and Diseases’ groupings for all condition categories with five or more guidelines. The condition categories, which achieved the lowest scores (ie, those with greater consideration of sex and/or gender dimensions), were cancer (3.43 average rating, n=7), musculoskeletal conditions (3.67 average rating, n=9) and mental health, behavioural and neurodevelopmental conditions (3.79 average rating, n=29). The condition categories, which scored highest (ie, those with less consideration of sex and/or gender dimensions), were digestive tract conditions (4.53 average rating, n=19), blood and immune system conditions (4.44 average rating, n=9) and skin conditions (4.4 average rating, n=5).

### Recommendations regarding women and girls

Reviewers identified that even when guidelines refer to a specific approach being needed to effectively treat female patients, they do not provide further information on these recommended approaches nor do they link to sex-specific guidance/materials. Overall, 178 guidelines (90%) did not link to relevant sex-specific guidance, and 54 (74%) of the 73 guidelines mentioning sex or gender dimensions of treatment and management did not. For example, recommendations for triage tools when managing pregnant women during major trauma (NG40) and specific predictive values for surgical interventions in pregnant patients with Crohn’s disease (NG129) state that specific considerations are needed for pregnant women, but do not include information on what those are.

### Use of terminology

Of the 197 guidelines, just 3 (1.5%) guidelines provided definitions of the sex and/or gender-related terminology used. An example of an included definition is ‘We have used the terms ‘men’ and ‘women’ in some recommendations on gender-related cancers, but they also apply to people who have changed or are in the process of changing gender, and who retain the relevant organs’ (NG12). Notably, the neonatal infection guideline (NG195) states that:

‘The guideline uses the terms ‘woman’ or ‘mother’ throughout. These should be taken to include people who do not identify as women but are pregnant or have given birth. Similarly, where the term ‘parents’ is used, this should be taken to include anyone who has the main responsibility for caring for a baby. We recognise that there are many different family arrangements.’

The introduction to the breast cancer guideline (NG101) states that:

‘This guideline uses specific, inclusive language to describe the population groups it covers (for example, women and pregnant people or trans and non-binary people) except when the evidence for the recommendation has not been reviewed and it is not certain from expert opinion whether it can cover more groups, or the evidence has been reviewed, but the information available for some groups at the time of development was too limited to make specific recommendations or only a very limited number of recommendations have been updated in direct response to new evidence or to reflect a change in practice.’

Of the 81 guidelines that referenced sex and/or gender outside of pregnancy and childbirth, 35 (43%) deployed sex- and gender-linked terminology inconsistently. Most commonly, this is because guidelines refer to gendered terms, such as ‘women’ and ‘men’ despite drawing data from databases where data were coded according to sex. 61 (75%) of these guidelines failed to consider sex and gender beyond the binary. Guidelines, which did account for non-binary or trans patients, made explicit recommendations on how to effectively manage all patients’ care. For instance, the cardiovascular disease guideline (NG238) states that ‘gender should be recorded as reported by the individual. If the individual discloses gender reassignment, they should be provided with cardiovascular disease risk calculations based on both genders and advised to discuss with their general practitioner, whose calculation is most appropriate for them as an individual’. All guidelines which effectively considered patients beyond the gender binary also provided explicit, accurate definitions of the terminology they use.

### Committee demographics

**Figure 4:**
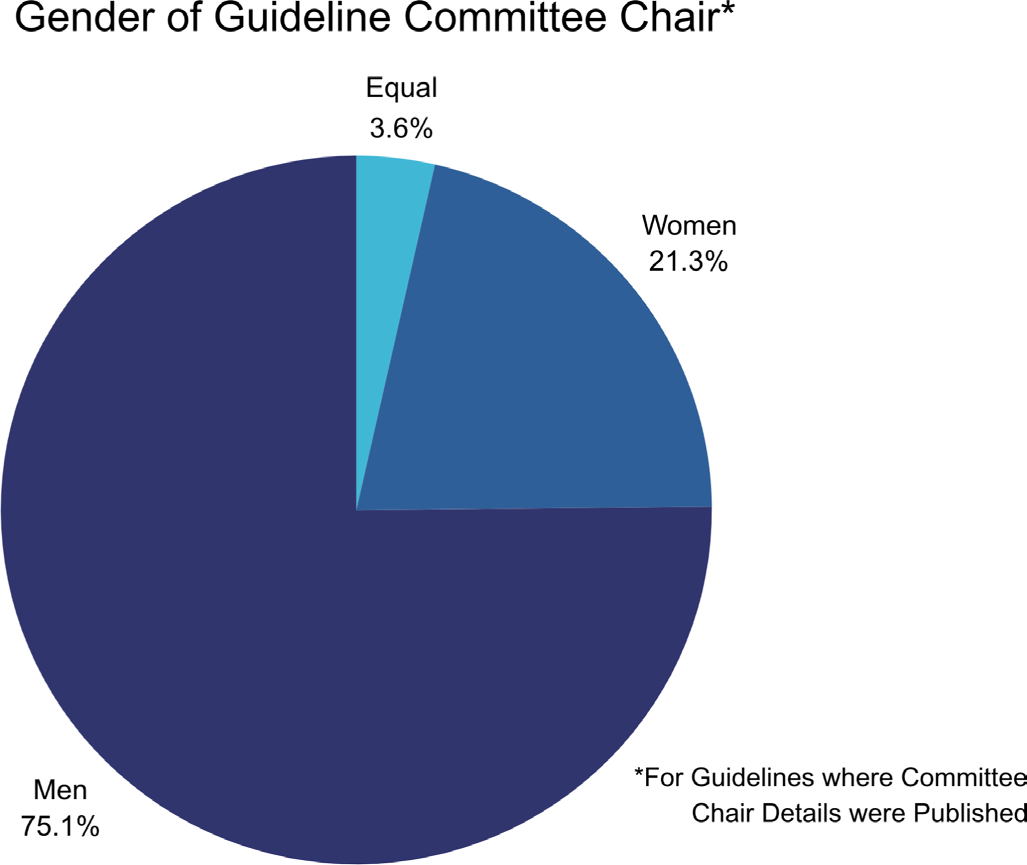
Gender of recent guideline committee chairs.

Of the 197 guidelines reviewed, 169 (86%) published the most recent committee chairs in their supporting documentation. 127/169 (75%) of the most recent guideline committees were chaired by men, while only 36/169 (21%) committees were chaired by women (figure 4). The remaining 4% of these committees were led jointly by women and men cochairs. Women were better represented in non-chair positions, where 48% of committee members and 65% of lay members were women.

We found that guidelines chaired by women achieved a higher rating for sex and gender consideration on average. Guidelines chaired by women were rated 3.92 on average, while committees chaired by men were rated 4.14 on average. According to our analysis, guidelines chaired by women were rated better than the overall average (4.07), while guidelines chaired by men were rated below average.

## Discussion

Results of this review offer a baseline assessment of how the UK’s clinical guidelines, developed and published by NICE, consider sex and gender dimensions of health and illness. Notably, only one guideline provided evidence-informed recommendations that support different or shared approaches for men and women, while 39% failed to account for sex and/or gender at all. Only 41% considered sex and/or gender dimensions beyond pregnancy and childbirth. This study demonstrates considerable gaps in NICE guidelines and highlights the need for urgent action to systematically ensure more comprehensive integration of sex and gender dimensions in current and future guidelines.

As in similar reviews, which identified inconsistent integration of sex and gender in Canadian^8^ and European (forthcoming) clinical practice guidelines, we found considerable heterogeneity in how clinical guidelines accounted for sex and gender. Such variation indicates that there is no standardised procedure for incorporating evidence on sex and gender differences or sex and gender terminology in NICE guidelines.

This study found that when evidence was known to be lacking for males, as in the case of breast cancer, this would be explicitly stated in the guidelines. By contrast, no such clarifications were identified when evidence was missing for female patients. These findings reflect the male default in medical research and clinical practice, whereby evidence or research from clinical trials involving male participants is presumed to extend to all patients rather than analyses performed to establish whether there may be sex and/or gender differences.^19^ This status quo risks minimising differences in men and in women by homogenising the participant group. New sex differences are likely to be discovered with a sex and gender intentional approach. For instance, sex-disaggregated use of the high-sensitivity cardiac troponin I assay with sex-specific thresholds increased diagnostic accuracy of myocardial injury in females by 42% and in males by 6%, showing how men and boys also stand to gain from a sex and gender intentional approach.^20^ It is likely that greater sex and gender disaggregation will disproportionately benefit groups who have historically been underrepresented in clinical research.^14^ Moreover, we found multiple instances where guidelines stated that clinical presentations, investigations or management may be different for women and/or females but did not provide information about what those differences would be. This trend is especially prevalent for pregnancy recommendations, as so many of the guidelines, which consider sex- and/or gender-specific management, do so solely in the context of pregnancy and breastfeeding. This lack of detailed recommendations and linked guidance speaks to a significant gap for pregnant and breastfeeding women as well as women in general.^21^

Management recommendations for women often referenced what procedures or medications should be avoided, typically focusing on what not to do, rather than providing alternative solutions. This supports the presence of a precautionary principle in care, which focuses on reducing medication-related risk rather than considering the opportunity cost of undertreated or untreated disease in pregnancy. It also makes decision-making in time-pressed primary care settings more challenging and puts patient safety at risk. It is notable that many guidelines that accounted for sex and gender did so in relation only to reproductive anatomy and reproductive systems. The only guideline to achieve a ‘Category 1’ rating is for fertility problems (CG156). In relation to females, 67% of the guidelines, which considered sex and/or gender, failed to do so beyond the context of reproduction. Many of the explicit sex differences cited in relation to males likewise focused on reproductive anatomy, such as considerations for male infertility or erectile dysfunction. These findings reflect the wider trend for ‘women’s health’ and ‘men’s health’ to be thought of as synonymous with sexual and reproductive health, with important sex and gender differences across all other diseases being neglected in clinical practice.^22^

Similarly, when sex and gender differences in medical conditions are mentioned in clinical guidelines, this is often done in reference to how that condition interacts with pivotal stages of the female lifecourse, such as menarche and menopause, or the multiple examples of pregnancy highlighted above. This evidence underlines the clear need for a lifecourse approach in medical research and practice that foregrounds how age and sex and/or gender intersect to shape women’s health. However, women’s health encompasses far more than life course transitions, and it is vital that the health of all women beyond these pivotal junctures, and crucially outside of fertility and childbearing, is adequately addressed through research and healthcare delivery.

For some NICE guidelines, recommendations had changed over time, from stating that there should be sex-specific diagnostic criteria or management practices to stating that the sexes should not be assessed differently. This trend was particularly prevalent across cardiovascular guidelines, for instance the acute coronary syndrome (NG1850) or recent onset chest pain (CG95) guidelines. These instances signify that in-depth study of sex and/or gender differences has been conducted and that new evidence has been used to update guidelines in relation to sex and/or gender. However, more widely, it is not possible to identify which NICE guidelines have paid attention to and taken up new evidence on sex and/or gender dimensions and which have not. Greater clarity is needed to convey whether sex-specific guidance is due to up-to-date evidence, a lack of evidence or existing evidence not having been integrated into guideline recommendations.

Only eight guidelines referred to trans, non-binary or intersex health, despite these groups having complex health needs and worse health outcomes than cisgender patients.^7^ This result reflects trends in the wider medical evidence base with poor consideration of sex and gender often rendering these groups invisible in study samples. It is vital that these patients, already known to be extremely underserved by healthcare systems and vulnerable to knowledge gaps among clinicians, are made visible in guidelines, as a necessary precursor to receiving correct and effective care. The purpose of guidelines is to set the standards of inclusion that are reflected in clinical practice. NICE has the opportunity to lead the sector in long overdue updates to emphasise equity, which will promote necessary inclusion and accuracy across medical practice.

The majority of guidelines failed to use sex- and/or gender-related terminology accurately or consistently, most often using gender-related terms, such as ‘women’ and ‘men’ when describing features that related to sex (where appropriate terminology would be ‘female’ or ‘male’). Very few guidelines included definitions for the sex and/or gender terminology used. Those which did include definitions tended to provide translations or explainers for non-inclusive language, rather than embedding more accurate and inclusive terminology. Consistent and inclusive use of sex and/or gender terminology in all guidelines is essential for improving clarity for guideline users as well as ensuring that guidelines are inclusive and scientifically accurate.

Comparison of the guidelines categorised by NICE as ‘Conditions and Diseases’ or ‘Health and Social Care Delivery’ revealed that Health and Social Care Delivery guidelines showed on average less consideration of sex and/or gender. While social care is often recognised as the ‘poor cousin of the NHS’ and UK Healthcare,^23^ it is key that sex and/or gender dimensions are considered not only in purely clinical contexts, but also in delivery aspects of healthcare services and the social care landscape. This is especially important, given the relatively more vulnerable status of those reliant on the social care system.^24^

Finally, we found that when guideline development was led by a woman chair, the guideline was more likely to account for sex and gender than if led by a man. While this relationship is not statistically significant, as there is a small sample size and no regression analysis was performed, there is an association here, which merits further investigation. Reviewers also found that, although there is a broadly even gender mix in committee memberships and lay representation, women are underrepresented in chair positions compared with men. This correlation could also suggest that fields that include more sex and gender differences in the evidence base, resulting in them being available for use in guidelines, are also more encouraging of championing women in leadership positions. Trends in guideline committee membership are reflected in healthcare leadership across the UK, where women are in the minority despite making up the majority of the health workforce.^23^ Our findings support evidence that including women in leadership positions to better reflect society’s gender mix is likely to improve equity and inclusion.^25^ Our findings underline that proactive efforts are needed to increase the number of women chairs in guideline expert committees, including removal of structural barriers to their participation. Trans, non-binary and intersex people in positions of power will also likely improve equity and efficacy of clinical care.

### Strengths and limitations

A strength of this study is that authors reviewed all NICE clinical guidelines, which resulted in comprehensive findings with relevance to all medical specialties. This allowed for comparison of sex and/or gender considerations between different body systems and specialties, an advantage compared with previous specialty-specific analyses, such as studies that have focused solely on cardiovascular disease^15^ or general internal medicine.^10^ Moreover, by studying NICE guidelines, which are collected in a central repository commissioned by the UK government, our study’s recommendations can be effectively targeted to UK policymakers, funders and health leaders to improve equity and representation in future guidelines and care, unlike studies spanning more dispersed geographies and healthcare systems.^10^

This study has some limitations. When recording whether guidelines accounted for sex and/or gender regarding pathophysiology, epidemiology, investigations and management, respectively, reviewers only recorded yes or no responses, rather than grading the quality or extent of this inclusion. This meant that, for analysis purposes, guidelines only briefly mentioning such aspects will have been treated the same as those with more expansive consideration. Relatedly, when recording the guideline’s consideration of sex and/or gender, reviewers did not denote whether inclusion was in relation to sex differences, gender differences or both. Nor did reviewers record whether sex and/or gender considerations related to men and/or male patients, women and/or female patients or trans, non-binary and intersex patients. Therefore, our analysis was unable to quantify which groups such considerations of sex and/or gender dimensions either benefited or neglected. Reviewers double reviewed all data where multiple interpretations were thought to be likely but did not double review all guideline data. When assessing the gender composition of guideline committees, reviewers used an online tool to categorise members based on whether their name was likely ‘male’ or ‘female’. This system means non-binary, trans or intersex representation could not be identified in the findings. When considering the potential relationship between guideline chair gender and category rating achieved, the number of guidelines reviewed was too low to be statistically significant nor were statistical tests performed.

### Future research

As a priority, considerable efforts are needed to generate evidence on sex and gender differences (and similarities) across all medical specialties and to identify existing evidence on this topic, which is not yet accounted for in clinical guidelines. International comparisons between clinical guidelines could serve as a fruitful way to start to better meet this need. Future research should also analyse whether considerations of sex differences, gender differences or both are accounted for within guidelines and, therefore, which gaps remain for evidence to fill. In addition, further studies could also consider the intersection of gender with other sociocultural factors (eg, race, ethnicity or age) in health leadership roles.

A systematic search for sex- and/or gender-specific evidence omitted from guidelines would permit the assessment of missed opportunities to appropriately include information about male and female patients in clinical guidelines.^9^ Future studies should also aim to record graded ratings for each aspect of sex and/or gender considerations, such as presentation, investigations or management, rather than solely recording their presence or absence. Future studies, perhaps those with greater numbers of guidelines or international comparators, could further investigate this relationship statistically.

### Implications

Our findings have wide implications for clinical practice and care, highlighting how gendered health inequities continue to be perpetuated. This study highlights the need for improved research into sex and/or gender dimensions of health and illness, an issue which emerging research funding policies, designed in partnership with the UK MESSAGE project, will address. A combination of funder policies with education and training will be key to progress this goal. Similarly, it is crucial that researchers and authors use the sex and gender equity in research ‘SAGER’ guidelines effectively when preparing papers for publication.^26^

This study highlights a clear need for NICE to establish a robust, standardised process for embedding sex- and/or gender-specific evidence in clinical guidelines. Likewise, standardised usage of sex and gender terminology will support equity and accuracy of guidelines. As such, future research should use uniform definitions of sex and gender, as these terms are frequently conflated in publications.^14^ We recognise that NICE already produces equality impact assessments, although these are stored in a separate location to the core clinical guidelines and are not integrated or linked to the guidelines themselves. We call on NICE to better integrate information on key differences, such as sex and gender, into the guidelines themselves, so relevant guidance is readily accessible to clinicians.

It is essential that NICE identifies where genuine evidence gaps persist, and where high-quality evidence is already published but has not been systematically integrated into relevant clinical guidelines. We call on NICE to undertake several initiatives to identify the existing key evidence on sex and/or gender dimensions across all clinical specialties. Recommended initiatives include a large-scale consultation, systematic review of the relevant literature and engagement with leading experts, all to identify existing key evidence. Based on the findings from these exercises, NICE should publish recommendations to highlight the most urgent data gaps. NICE should also establish a mechanism to regularly embed relevant sex and/or gender disaggregated evidence in guidelines and flag this to users. In particular, NICE should include a mandatory specific question on sex and/or gender in all future guideline consultations published. We further encourage NICE to expand their equity responsibilities beyond sex and gender to consider race, ethnicity, age and how these intersect.

In summary, this systematic review demonstrates considerable gaps in how NICE clinical guidelines account for sex and gender dimensions of health and illness, suggesting NICE has not accounted for these critical dimensions throughout guideline development in a standardised way. It also highlights that increased representation of women among guideline development committee chairs is likely to improve guidelines’ consideration of sex and gender dimensions. As a single body dedicated to clinical guideline development—a rarity in most health systems—NICE has a unique opportunity to pioneer robust and consistent mechanisms to ensure and promote more accurate, inclusive and evidence-based healthcare recommendations that can reduce health inequities and improve patient outcomes for all. The NHS has a long-term goal to provide personalised medicine,^27^ but until these critical aspects of evidence generation and clinical management are accounted for, providing intersectional and personalised healthcare remains a distant possibility.

This article calls on NICE to invest in addressing these critical gaps, including running a consultation to identify and embed key evidence on sex and/or gender dimensions across all guidelines wherever relevant.

## Supplementary material

10.1136/bmjph-2024-002510 online supplemental file 1

## Data Availability

All data relevant to the study are included in the article or uploaded as supplementary information.
