# Supplementary material for: Are sex and gender dimensions accounted for in NICE guidelines? A systematic review of 223 clinical guidelines
Source: BMJ Public Health. 2025 Jul 17;3(2):e002510. doi: 10.1136/bmjph-2024-002510 (PMC12273117; doi:10.1136/bmjph-2024-002510)
Supplement: online supplemental file 1 [file bmjph-3-2-s001.pdf]

NICE Clincal Guidelines Sex and Gender Review - Summary of Key Data Collected

Title	Number	Publication date	Last ipdated	Assessing a sex-specific condition?	Body system	Sex-related and/or gender-related terminology used?	Rating category achieved	Chair gender (most recent committee)
Abdominal aortic aneurysm: diagnosis and management	NG156	19-Mar-20	19-Mar-20	No	Cardiovascular conditions	Yes	3	Man
Acute kidney injury: prevention, detection and management	NG148	18-Dec-19	28-Sep-23	No	Kidney Conditions	No	5	Not Listed
Age-related macular degeneration	NG82	23-Jan-18	23-Jan-18	No	Eye Conditions	No	5	Man
Alcohol-use disorders: diagnosis and management of physical complications	CG100	02-Jun-10	12-Apr-17	No	Liver conditions	Yes	4	Woman
Alcohol-use disorders: diagnosis, assessment and management of harmful drinking (high-risk drinking) and alcohol dependence	CG115	23-Feb-11	21-Oct-14	No	Liver conditions	Yes	3	Man
Asthma: diagnosis, monitoring and chronic asthma management	NG80	29-Nov-17	22-Mar-21	No	Respiratory conditions	No	[illegible]	[illegible]
Bladder cancer: diagnosis and management	NG2	25-Feb-15	25-Feb-15	No	Urological conditions	Yes	3	Not Listed
Cataracts in adults: management	NG77	26-Oct-17	26-Oct-17	No	Eye Conditions	No	5	Man
Chronic kidney disease: assessment and management	NG203	25-Aug-21	24-Nov-21	No	Kidney Conditions	Yes	2	Man
Chronic obstructive pulmonary disease in over 16s: diagnosis and management	NG115	05-Dec-18	26-Jul-19	No	Respiratory conditions	No	5	Man
Cystic fibrosis: diagnosis and management	NG78	25-Oct-17	25-Oct-17	No	Respiratory conditions	Yes	3	Man
Dental checks: intervals between oral health reviews	CG19	27-Oct-04	27-Oct-04	No	Oral and Dental Health	Yes	4	Woman
Drug allergy: diagnosis and management	CG183	03-Sep-14	03-Sep-14	No	Blood and immune system conditions	No	5	Not Listed
Drug misuse in over 16s: opioid detoxification	CG52	25-Jul-07	25-Jul-07	No	Mental health, behavioural and neurodevelopmental conditions	Yes	4	Not Listed
Drug misuse in over 16s: psychosocial interventions	CG51	25-Jul-07	25-Jul-07	No	Mental health, behavioural and neurodevelopmental conditions	No	5	Man
Falls in older people: assessing risk and prevention	CG161	12-Jun-13	12-Jun-13	No	Injuries, accidents and wounds	Yes	4	Man
Familial hypercholesterolaemia: identification and management	CG71	27-Aug-08	04-Oct-19	No	Cardiovascular conditions	Yes	3	Man
Glaucoma: diagnosis and management	NG81	01-Nov-17	26-Jan-22	No	Eye conditions	No	5	Man
Hearing loss in adults: assessment and management	NG98	21-Jun-18	02-Oct-23	No	Ear, nose and throat conditions	No	5	Woman
Hypertension in adults: diagnosis and management	NG136	28-Aug-19	21-Nov-23	No	Cardiovascular conditions	Yes	3	Man
Idiopathic pulmonary fibrosis in adults: diagnosis and management	CG163	12-Jun-13	23-May-17	No	Respiratory conditions	No	5	Man
Intravenous fluid therapy in children and young people in hospital	NG29	09-Dec-15	11-Jun-20	No	Health and social care delivery	Yes	4	Not Listed
Multimorbidity: clinical assessment and management	NG56	21-Sep-16	21-Sep-16	No	Multiple long-term conditions	Yes	2	Man
Nutrition support for adults: oral nutrition support, enteral tube feeding and parenteral nutrition	CG32	22-Feb-06	04-Aug-17	No	Diabetes and other endocrinal, nutritional and metabolic conditions	Yes	4	Man
Obstructive sleep apnoea/hypopnoea syndrome and obesity hypoventilation syndrome in over 16s	NG202	20-Aug-21	20-Aug-21	No	Sleep and sleep conditions	Yes	3	Man
Organ donation for transplantation: improving donor identification and consent rates for deceased organ donation	CG135	12-Dec-11	21-Dec-16	No	Health and social care delivery	No	5	Not Listed
Patient experience in adult NHS services: improving the experience of care for people using adult NHS services	CG138	24-Feb-12	17-Jun-21	No	Health and social care delivery	Yes	4	Not Listed
Perioperative care in adults	NG180	19-Aug-20	19-Aug-20	No	Health and social care delivery	No	5	Man
Peripheral arterial disease: diagnosis and management	CG147	08-Aug-12	11-Dec-20	No	Cardiovascular conditions	No	5	Not Listed
Physical health of people in prison	NG57	02-Nov-16	02-Nov-16	No	Health and social care delivery	Yes	2	Man
Pneumonia in adults: diagnosis and management	CG191	03-Dec-14	31-Oct-23	No	Respiratory conditions	Yes	4	Not Listed
Pressure ulcers: prevention and management	CG179	23-Apr-14	23-Apr-14	No	Skin Conditions	No	5	Not Listed
Psoriasis: assessment and management	CG153	24-Oct-12	01-Sep-17	No	Skin Conditions	Yes	4	Not Listed
Rehabilitation after critical illness in adults	CG83	25-Mar-09	25-Mar-09	No	Health and social care delivery	No	5	Not Listed
Renal and ureteric stones: assessment and management	NG118	08-Jan-19	08-Jan-19	No	Kidney conditions	Yes	4	Man
Renal replacement therapy and conservative management	NG107	03-Oct-18	03-Oct-18	No	Kidney conditions	Yes	4	Woman
Routine preoperative tests for elective surgery	NG45	05-Apr-16	05-Apr-16	No	Health and social care delivery	Yes	4	Man
Sedation in under 19s: using sedation for diagnostic and therapeutic procedures	CG112	15-Dec-10	15-Dec-10	No	Health and social care delivery	No	5	Man
Shared decision making	NG197	17-Jun-21	17-Jun-21	No	Health and social care delivery	No	5	Woman
Specialist neonatal respiratory care for babies born preterm	NG124	03-Apr-19	03-Apr-19	No	Respiratory conditions	Yes	3	Woman
Suspected acute respiratory infection in over 16s: assessment at first presentation and initial management	NG237	31-Oct-23	16-Nov-23	No	Respiratory conditions	Yes	4	Woman
Tinnitus: assessment and management	NG155	11-Mar-20	11-Mar-20	No	Ear, nose and throat conditions	No	5	Woman
Tuberculosis	NG33	13-Jan-16	16-Feb-24	No	Respiratory conditions	Yes	4	Man
Varicose veins: diagnosis and management	CG168	24-Jul-13	24-Jul-13	No	Cardiovascular conditions	Yes	4	Man
Venous thromboembolic diseases: diagnosis, management and thrombophilia testing	NG158	26-Mar-20	02-Aug-23	No	Cardiovascular conditions	Yes	4	Woman
Venous thromboembolism in over 16s: reducing the risk of hospital-acquired deep vein thrombosis or pulmonary embolism	NG89	21-Mar-18	13-Aug-19	No	Cardiovascular conditions	Yes	3	Man
Acute upper gastrointestinal bleeding in over 16s: management	CG141	13-Jun-12	25-Aug-16	No	Digestive tract conditions	No	5	Man
Acutely ill adults in hospital: recognising and responding to deterioration	CG50	25-Jul-07	25-Jul-07	No	Health and social care delivery	No	5	Woman
Anaphylaxis: assessment and referral after emergency treatment	CG134	14-Dec-11	24-Aug-20	No	Blood and immune system conditions	No	5	Man
Barrett's oesophagus and stage 1 oesophageal adenocarcinoma: monitoring and management	NG231	08-Feb-23	08-Feb-23	No	Digestive tract conditions	No	5	Man
Cancer of the upper aerodigestive tract: assessment and management in people aged 16 and over	NG36	10-Feb-16	06-Jun-18	No	Digestive tract conditions	No	5	Man
Cirrhosis in over 16s: assessment and management	NG50	06-Jul-16	08-Sep-23	No	Liver conditions	Yes	2	Man
Coeliac disease: recognition, assessment and management	NG20	02-Sep-15	02-Sep-15	No	Digestive tract conditions	No	5	Man
Colorectal cancer	NG151	29-Jan-20	15-Dec-21	No	Digestive tract conditions	No	5	Man
Colorectal cancer prevention: colonoscopic surveillance in adults with ulcerative colitis, Crohn's disease or adenomas	CG118	23-Mar-11	20-Sep-22	No	Digestive tract conditions	No	5	Man
Constipation in children and young people: diagnosis and management	CG99	26-May-10	13-Jul-17	No	Digestive tract conditions	No	5	Woman
Crohn's disease: management	NG129	03-May-19	03-May-19	No	Digestive tract conditions	Yes	4	Woman
Diverticular disease: diagnosis and management	NG147	27-Nov-19	27-Nov-19	No	Digestive tract conditions	Yes	3	Man
Emergency and acute medical care in over 16s: service delivery and organisation	NG94	28-Mar-18	28-Mar-18	No	Health and social care delivery	No	5	Man
Faecal incontinence in adults: management	CG49	27-Jun-07	27-Jun-07	No	Digestive tract conditions	Yes	3	Woman
Gallstone disease: diagnosis and management	CG188	29-Oct-14	29-Oct-14	No	Digestive tract conditions	No	5	Man
Gastro-oesophageal reflux disease and dyspepsia in adults: investigation and management	CG184	03-Sep-14	18-Oct-19	No	Digestive tract conditions	No	5	Man

Gastro-oesophageal reflux disease in children and young people: diagnosis and management	NG1	14-Jan-15	09-Oct-19	No	Digestive tract conditions	No	5	Man
Hypothermia: prevention and management in adults having surgery	CG65	23-Apr-08	14-Dec-16	No	Health and social care delivery	No	5	Man
Intravenous fluid therapy in adults in hospital	CG174	10-Dec-13	05-May-17	No	Health and social care delivery	No	5	Man
Irritable bowel syndrome in adults: diagnosis and management	CG61	23-Feb-08	04-Apr-17	No	Digestive tract conditions	Yes	3	equal
Major trauma: assessment and initial management	NG39	17-Feb-16	17-Feb-16	No	Injuries, accidents and wounds	No	5	Man
Medicines adherence: involving patients in decisions about prescribed medicines and supporting adherence	CG76	28-Jan-09	28-Jan-09	No	Health and social care delivery	No	5	Man
Medicines associated with dependence or withdrawal symptoms: safe prescribing and withdrawal management for adults	NG215	20-Apr-22	20-Apr-22	No	Health and social care delivery	Yes	4	Woman
Medicines optimisation: the safe and effective use of medicines to enable the best possible outcomes	NG5	04-Mar-15	04-Mar-15	No	Health and social care delivery	No	5	Man
Non-alcoholic fatty liver disease (NAFLD): assessment and management	NG49	06-Jul-16	06-Jul-16	No	Liver conditions	No	5	Man
Oesophago-gastric cancer: assessment and management in adults	NG83	24-Jan-18	04-Jul-23	No	Digestive tract conditions	No	5	Woman
Pancreatic cancer in adults: diagnosis and management	NG85	07-Feb-18	07-Feb-18	No	Digestive tract conditions	No	5	Man
Pancreatitis	NG104	05-Sep-18	16-Dec-20	No	Digestive tract conditions	No	5	Man
Ulcerative colitis: management	NG130	03-May-19	03-May-19	No	Digestive tract conditions	Yes	3	Woman
Acne vulgaris: management	NG198	25-Jun-21	7-Dec-23	No	Skin Conditions	Yes	4	Woman
Advanced breast cancer: diagnosis and treatment	CG81	23-Feb-09	16-Aug-17	No	Cancer	Yes	3	Not Listed
Atopic eczema in under 12s: diagnosis and management	CG57	12-Dec-07	7-Jun-23	No	Skin Conditions	No	5	Woman
Babies, children and young people's experience of healthcare	NG204	25-Aug-21	25-Aug-21	No	Health and social care delivery	Yes	4	Woman
Bedwetting in under 19s	CG111	27-Oct-10	27-Oct-10	No	Neurological conditions	Yes	4	Not Listed
Brain tumours (primary) and brain metastases in over 16s	NG99	11-Jul-18	29-Jan-21	No	Neurological conditions	No	5	Man
Bronchiolitis in children: diagnosis and management	NG9	1-Jun-15	9-Aug-21	No	Respiratory conditions	No	5	Man
Child maltreatment: when to suspect maltreatment in under 18s	CG89	22-Jul-09	9-Oct-17	No	Injuries, accidents and wounds	Yes	4	Woman
Children's attachment: attachment in children and young people who are adopted from care, in care or at high risk of going into care	NG26	25-Nov-15	25-Nov-15	No	Health and social care delivery	No	5	Man
Developmental follow-up of children and young people born preterm	NG72	9-Aug-17	9-Aug-17	No	Fertility, pregnancy and childbirth	Yes	3	Woman
Diarrhoea and vomiting caused by gastroenteritis in under 5s: diagnosis and management	CG84	22-Apr-09	22-Apr-09	No	Digestive tract conditions	No	5	Man
Early and locally advanced breast cancer: diagnosis and management	NG101	18-Jul-18	16-Jan-24	No	Cancer	Yes	3	Woman
End of life care for infants, children and young people with life-limiting conditions: planning and management	NG61	7-Dec-16	25-Jul-19	No	Health and social care delivery	No	5	equal
Faltering growth: recognition and management of faltering growth in children	NG75	27-Sep-17	27-Sep-17	No	Diabetes and other endocrinal, nutritional and metabolic conditions	Yes	4	Man
Familial breast cancer: classification, care and managing breast cancer and related risks in people with a family history of breast cancer	CG164	25-Jun-13	14-Nov-23	No	Cancer	Yes	4	Woman
Fever in under 5s: assessment and initial management	NG143	7-Nov-19	26-Nov-21	No	Infections	No	5	Man
Food allergy in under 19s: assessment and diagnosis	CG116	23-Feb-11	23-Feb-11	No	Blood and immune system conditions	No	5	Man
Haematological cancers: improving outcomes	NG47	25-May-16	25-May-16	No	Blood and immune system conditions	No	5	Man
Jaundice in newborn babies under 28 days	CG98	19-May-10	31-Oct-23	No	Blood and immune system conditions	Yes	4	Woman
Joint replacement (primary): hip, knee and shoulder	NG157	4-Jun-20	4-Jun-20	No	Musculoskeletal conditions	No	5	Man
Melanoma: assessment and management	NG14	29-Jul-15	27-Jul-22	No	Skin conditions	Yes	4	Man
Metastatic malignant disease of unknown primary origin in adults: diagnosis and management	CG104	26-Jul-10	26-Apr-23	No	Cancer	Yes	2	Man
Myeloma: diagnosis and management	NG35	10-Feb-16	25-Oct-18	No	Blood and immune system conditions	Yes	3	Not Listed
Neonatal infection: antibiotics for prevention and treatment	NG195	20-Apr-21	19-Mar-24	No	Infections	Yes	4	Man
Neonatal parenteral nutrition	NG154	26-Feb-20	26-Feb-20	No	Infants and neonates	No	5	Man
Non-Hodgkin's lymphoma: diagnosis and management	NG52	20-Jul-16	20-Jul-16	No	Cancer	No	5	Man
Otitis media with effusion in under 12s	NG233	30-Aug-23	30-Aug-23	No	Ear, nose and throat conditions	No	5	Woman
Sickle cell disease: managing acute painful episodes in hospital	CG143	27-Jun-12	27-Jun-12	No	Blood and immune system conditions	Yes	4	Man
Suspected cancer: recognition and referral	NG12	23-Jun-15	2-Oct-23	No	Cancer	Yes	3	Woman
Care of dying adults in the last days of life	NG31	16-Dec-15	16-Dec-15	No	Health and social care delivery	No	5	Man
Diabetes (type 1 and type 2) in children and young people: diagnosis and management	NG18	1-Aug-15	11-May-23	No	Diabetes and other endocrinal, nutritional and metabolic conditions	No	5	Man
Diabetic foot problems: prevention and management	NG19	26-Aug-15	11-Oct-19	No	Diabetes and other endocrinal, nutritional and metabolic conditions	Yes	4	Man
End of life care for adults: service delivery	NG142	16-Oct-19	16-Oct-19	No	Health and social care delivery	No	5	Woman
Healthcare-associated infections: prevention and control in primary and community care	CG139	28-Mar-12	15-Feb-17	No	Infections	Yes	3	Not Listed
Hepatitis B (chronic): diagnosis and management	CG165	26-Jun-13	20-Oct-17	No	Infections	Yes	2	Man
Hyperparathyroidism (primary): diagnosis, assessment and initial management	NG132	23-May-19	23-May-19	No	Diabetes and other endocrinal, nutritional and metabolic conditions	Yes	4	Man
Lyme disease	NG95	11-Apr-18	17-Oct-18	No	Infections	No	5	Man
Meningitis (bacterial) and meningococcal disease: recognition, diagnosis and management	NG240	19-Mar-24	19-Mar-24	No	Infections	No	5	Man
Myalgic encephalomyelitis (or encephalopathy)/chronic fatigue syndrome: diagnosis and management	NG206	29-Oct-21	29-Oct-21	No	ME/CFS	Yes	4	Man
Neuropathic pain in adults: pharmacological management in non-specialist settings	CG173	20-Nov-13	22-Sep-20	No	Chronic and neuropathic pain	Yes	4	Not Listed
Neutropenic sepsis: prevention and management in people with cancer	CG151	19-Sep-12	19-Sep-12	No	Blood and immune system conditions	No	5	Man
Obesity prevention	CG43	13-Dec-06	13-Mar-15	No	Diabetes and other endocrinal, nutritional and metabolic conditions	Yes	4	Man
Obesity: identification, assessment and management	CG189	27-Nov-14	26-Jul-23	No	Diabetes and other endocrinal, nutritional and metabolic conditions	Yes	4	equal
Palliative care for adults: strong opioids for pain relief	CG140	23-May-12	03-Aug-16	No	Health and social care delivery	No	5	Man
Spinal metastases and metastatic spinal cord compression	NG234	06-Sep-23	06-Sep-23	No	Neurological conditions	Yes	4	Man
Stroke and transient ischaemic attack in over 16s: diagnosis and initial management	NG128	01-May-19	13-Apr-22	No	Neurological conditions	No	5	Man
Stroke rehabilitation in adults	NG236	18-Oct-23	18-Oct-23	No	Neurological conditions	No	5	Man
Subarachnoid haemorrhage caused by a ruptured aneurysm: diagnosis and management	NG228	23-Nov-22	23-Nov-22	No	Cardiovascular conditions	Yes	3	Man
Surgical site infections: prevention and treatment	NG125	11-Apr-19	19-Aug-20	No	Infections	Yes	4	Man
Suspected sepsis: recognition, diagnosis and early management	NG51	13-Jul-16	19-Mar-24	No	Infections	Yes	2	equal
Thyroid cancer: assessment and management	NG230	19-Dec-22	19-Dec-22	No	Cancer	Yes	4	Man
Thyroid disease: assessment and management	NG145	20-Nov-19	12-Oct-23	No	Diabetes and other endocrinal, nutritional and metabolic conditions	Yes	3	Woman
Type 1 diabetes in adults: diagnosis and management	NG17	26-Aug-15	17-Aug-22	No	Diabetes and other endocrinal, nutritional and metabolic conditions	Yes	4	Man

Type 2 diabetes in adults: management	NG28	02-Dec-15	29-Jun-22	No	Diabetes and other endocrinal, nutritional and metabolic conditions	Yes	4	Man
Urinary incontinence in neurological disease: assessment and management	CG148	08-Aug-12	02-Oct-23	No	Neurological conditions	Yes	4	Man
Urinary tract infection in under 16s: diagnosis and management	NG224	27-Jul-22	27-Jul-22	No	Urological conditions	Yes	4	Woman
Vitamin B12 deficiency in over 16s: diagnosis and management	NG239	06-Mar-24	06-Mar-24	No	Blood and immune system conditions	Yes	4	Man
Antisocial behaviour and conduct disorders in children and young people: recognition and management	CG158	27-Mar-13	19-Apr-17	No	Mental health, behavioural and neurodevelopmental conditions	Yes	3	Not Listed
Antisocial personality disorder: prevention and management	CG77	28-Jan-09	27-Mar-13	No	Mental health, behavioural and neurodevelopmental conditions	Yes	3	Man
Attention deficit hyperactivity disorder: diagnosis and management	NG87	14-Mar-18	13-Sep-19	No	Mental health, behavioural and neurodevelopmental conditions	Yes	3	Woman
Autism spectrum disorder in adults: diagnosis and management	CG142	27-Jun-12	14-Jun-21	No	Mental health, behavioural and neurodevelopmental conditions	Yes	4	Man
Autism spectrum disorder in under 19s: recognition, referral and diagnosis	CG128	28-Sep-11	20-Dec-17	No	Mental health, behavioural and neurodevelopmental conditions	Yes	3	Woman
Autism spectrum disorder in under 19s: support and management	CG170	28-Aug-13	14-Jun-21	No	Mental health, behavioural and neurodevelopmental conditions	Yes	3	Not Listed
Bipolar disorder: assessment and management	CG185	24-Sep-14	21-Dec-23	No	Mental health, behavioural and neurodevelopmental conditions	Yes	4	Not Listed
Borderline personality disorder: recognition and management	CG78	28-Jan-09	28-Jan-09	No	Mental health, behavioural and neurodevelopmental conditions	Yes	3	Not Listed
Challenging behaviour and learning disabilities: prevention and interventions for people with learning disabilities whose behaviour challenges	NG11	29-May-15	29-May-15	No	Health and social care delivery	No	5	Not Listed
Coexisting severe mental illness (psychosis) and substance misuse: assessment and management in healthcare settings	CG120	23-Mar-11	23-Mar-11	No	Mental health, behavioural and neurodevelopmental conditions	No	5	Man
Delirium: prevention, diagnosis and management in hospital and long-term care	CG103	28-Jul-10	18-Jan-23	No	Mental health, behavioural and neurodevelopmental conditions	No	5	Man
Dementia: assessment, management and support for people living with dementia and their carers	NG97	20-Jun-18	20-Jun-18	No	Mental health, behavioural and neurodevelopmental conditions	Yes	4	Man
Depression in adults with a chronic physical health problem: recognition and management	CG91	28-Oct-09	28-Oct-09	No	Mental health, behavioural and neurodevelopmental conditions	No	5	Man
Depression in adults: treatment and management	NG222	29-Jun-22	29-Jun-22	No	Mental health, behavioural and neurodevelopmental conditions	Yes	3	Man
Depression in children and young people: identification and management	NG134	25-Jun-19	25-Jun-19	No	Mental health, behavioural and neurodevelopmental conditions	No	5	Woman
Eating disorders: recognition and treatment	NG69	23-May-17	16-Dec-20	No	Mental health, behavioural and neurodevelopmental conditions	Yes	4	Man
Fertility problems: assessment and treatment	CG156	20-Feb-13	06-Sep-17	No	Fertility, pregnancy and childbirth	Yes	1	Woman
Generalised anxiety disorder and panic disorder in adults: management	CG113	26-Jan-11	15-Jun-20	No	Mental health, behavioural and neurodevelopmental conditions	Yes	3	Not Listed
Mental health of adults in contact with the criminal justice system	NG66	21-Mar-17	21-Mar-17	No	Mental health, behavioural and neurodevelopmental conditions	Yes	2	Man
Mental health problems in people with learning disabilities: prevention, assessment and management	NG54	14-Sep-16	14-Sep-16	No	Mental health, behavioural and neurodevelopmental conditions	No	5	Not Listed
Obsessive-compulsive disorder and body dysmorphic disorder: treatment	CG31	29-Nov-05	29-Nov-05	No	Mental health, behavioural and neurodevelopmental conditions	No	5	Not Listed
Post-traumatic stress disorder	NG116	05-Dec-18	05-Dec-18	No	Mental health, behavioural and neurodevelopmental conditions	Yes	4	Man
Psychosis and schizophrenia in adults: prevention and management	CG178	12-Feb-14	01-Mar-14	No	Mental health, behavioural and neurodevelopmental conditions	Yes	4	Woman
Psychosis and schizophrenia in children and young people: recognition and management	CG155	23-Jan-13	26-Oct-16	No	Mental health, behavioural and neurodevelopmental conditions	Yes	4	Not Listed
Rehabilitation for adults with complex psychosis	NG181	19-Aug-20	19-Aug-20	No	Mental health, behavioural and neurodevelopmental conditions	Yes	3	Woman
Self-harm: assessment, management and preventing recurrence	NG225	07-Sep-22	07-Sep-22	No	Mental health, behavioural and neurodevelopmental conditions	Yes	3	Man
Service user experience in adult mental health: improving the experience of care for people using adult NHS mental health services	CG136	14-Dec-11	14-Dec-11	No	Mental health, behavioural and neurodevelopmental conditions	Yes	3	Not Listed
Social anxiety disorder: recognition, assessment and treatment	CG159	22-May-13	22-May-13	No	Mental health, behavioural and neurodevelopmental conditions	No	5	Man
Violence and aggression: short-term management in mental health, health and community settings	NG10	28-May-15	28-May-15	No	Mental health, behavioural and neurodevelopmental conditions	Yes	3	Not Listed
Hip fracture: management	CG124	22-6-11	06 Jan -23	No	Musculoskeletal conditions	Yes	4	Man
Osteoporosis: assessing the risk of fragility fracture	CG146	8-8-12	7 Feb 17	No	Musculoskeletal conditions	Yes	2	Man
Osteoarthritis in over 16s: diagnosis and management	NG226	19 Oct 22	19 Oct 22	No	Musculoskeletal conditions	Yes	3	Man
Gout: diagnosis and management	NG219	9 Jun 22	9 Jun 22	No	Musculoskeletal conditions	Yes	3	Man
Rheumatoid arthritis in adults: management	NG100	11 Jul 18	12 Oct 20	No	Musculoskeletal conditions	Yes	3	Man
Spondyloarthritis in over 16s: diagnosis and management	NG65	28 Feb 17	2 Jun 17	No	Musculoskeletal conditions	Yes	3	Man
Chronic pain (primary and secondary) in over 16s: assessment of all chronic pain and management of chronic primary pain	NG193	7 Apr 21	7 Apr 21	No	Chronic and neuropathic pain	Yes	4	Man
Low back pain and sciatica in over 16s: assessment and management	NG59	30 Nov 16	11 Dec 20	No	Musculoskeletal conditions	No	5	Man
Head injury: assessment and early management	NG232	18-May-23	18-May-23	No	Injuries, accidents and wounds	Yes	4	Man
Fractures (complex): assessment and management	NG37	17-Feb-16	23-Nov-22	No	Injuries, accidents and wounds	No	5	Man
Rehabilatation after traumatic injury	NG211	18-Jan-22	18-Jan-22	No	Injuries, accidents and wounds	Yes	4	Man
Major trauma: service delivery	NG40	17-Feb-16	17-Feb-16	No	Injuries, accidents and wounds	Yes	4	Man
Fractures (non-complex): assessment and management	NG38	17-Feb-16	17-Feb-16	No	Musculoskeletal conditions	No	5	Man
Spinal injury: assessment and initial management	NG41	17-Feb-16	1-May-21	No	Neurological conditions	Yes	4	Man
Blood Transfusion	NG24	18-Nov-15	Aug-23	No	Injuries, accidents and wounds	Yes	4	Man
Cardiovascular disease: risk assessment and reduction, including lipid modification	NG238	14-Dec-23	14-Dec-23	No	Cardiovascular conditions	Yes	4	equal
Acute Coronary Syndromes	NG185	18-Nov-20	Dec-21	No	Cardiovascular conditions	Yes	4	Woman
Recent-onset chest pain of suspected cardiac origin: assessment and diagnosis	CG95	24-Mar-10	30-Nov-16	No	Cardiovascular conditions	Yes	4	Man
Acute heart failure: diagnosis and management	CG187	08-Oct-14	17-Nov-21	No	Cardiovascular conditions	No	5	Man
Chronic heart failure in adults: diagnosis and management	NG106	12-Sep-18	Nov-21	No	Cardiovascular conditions	Yes	4	Man
Atrial fibrillation: diagnosis and management	NG196	27-Apr-21	30-Jun-21	No	Cardiovascular conditions	Yes	4	Man
Heart valve disease presenting in adults: investigation and management	NG208	17-Nov-21	Dec-21	No	Cardiovascular conditions	Yes	4	Man
Prophylaxis against infective endocarditis: antimicrobial prophylaxis against infective endocarditis in adults and children undergoing interventional procedures	CG64	17-Mar-08	08-Jul-16	No	Cardiovascular conditions	No	5	equal
Stable angina: management	CG126	23-Jul-11	25-Aug-16	No	Cardiovascular conditions	Yes	4	Man
Suspected neurological conditions: recognition and referral	NG127	01-May-24	02/10/2023	No	Neurological conditions	Yes	2	Man
Epilepsies in children, young people and adults	NG217	27-Apr-22	27-Apr-22	No	Neurological conditions	Yes	4	Man
Cannabis-based medicinal products	NG144	11-Nov-19	22-Mar-21	No	Neurological conditions	Yes	4	Man
Headaches in over 12s: diagnosis and management	CG150	19-Sep-12	17-Dec-21	No	Neurological conditions	Yes	3	Man
Motor neurone disease: assessment and management	NG42	24-Feb-16	23-Jul-19	No	Neurological conditions	Yes	3	Man

Multiple sclerosis in adults: management	NG220	22-Jun-22	22-Jun-22	No	Neurological conditions	Yes	3	Man
Parkinson's disease in adults	NG71	19-Jul-17	01-May-22	No	Neurological conditions	Yes	4	Man
Cerebral palsy in adults	NG119	15-Jan-19	01-May-22	No	Neurological conditions	Yes	4	Man
Cerebral palsy in under 25s: assessment and management	NG62	25-Jan-17	Oct-19	No	Neurological conditions	Yes	4	Man
Spasticity in under 19s: management	CG145	25-Jul-12	29-Nov-16	No	Neurological conditions	No	5	Man
Transient loss of consciousness ('blackouts') in over 16s	CG109	25-Aug-10	21-Nov-23	No	Neurological conditions	No	5	Man
Lung cancer: diagnosis and management	NG122	28-Mar-19	08-Mar-24	No	Respiratory conditions	Yes	3	Man
Abortion care	NG140	25 September 201	25-Sep-19	Yes	-	-	-	-
Antenatal and postnatal mental health: clinical management and service guidance	CG192	17 December 2014	11-Feb-20	Yes	-	-	-	-
Antenatal care	NG201	19 August 2021	19-Aug-21	Yes	-	-	-	-
Caesarean birth	NG192	31 March 2021	30-Jan-24	Yes	-	-	-	-
Diabetes in pregnancy: management from preconception to the postnatal period	NG3	25 February 2015	16-Dec-20	Yes	-	-	-	-
Donor milk banks: service operation	CG93	24 February 2010	24-Feb-10	Yes	-	-	-	-
Ectopic pregnancy and miscarriage: diagnosis and initial management	NG126	17 April 2019	23-Aug-23	Yes	-	-	-	-
Endometriosis: diagnosis and management	NG73	6 September 2017	16-Apr-24	Yes	-	-	-	-
Fetal monitoring in labour	NG229	14 December 2022	14-Dec-22	Yes	-	-	-	-
Heavy menstrual bleeding: assessment and management	NG88	14 March 2018	24-May-21	Yes	-	-	-	-
Hypertension in pregnancy: diagnosis and management	NG133	25 June 2019	17-Apr-23	Yes	-	-	-	-
Inducing labour	NG207	4 November 2021	04-Nov-21	Yes	-	-	-	-
Intrapartum care	NG235	29 September 202	29-Sep-23	Yes	-	-	-	-
Intrapartum care for women with existing medical conditions or obstetric complications and their babies	NG121	6 March 2019	25-Apr-19	Yes	-	-	-	-
Long-acting reversible contraception	CG30	26 October 2005	02-Jul-19	Yes	-	-	-	-
Lower urinary tract symptoms in men: management	CG97	23 May 2010	03-Jun-15	Yes	-	-	-	-
Menopause: diagnosis and management	NG23	12 November 2015	05-Dec-19	Yes	-	-	-	-
Ovarian cancer: identifying and managing familial and genetic risk	NG241	21 March 2024	21-Mar-24	Yes	-	-	-	-
Ovarian cancer: recognition and initial management	CG122	27 April 2011	02-Oct-23	Yes	-	-	-	-
Pelvic floor dysfunction: prevention and non-surgical management	NG210	9 December 2021	09-Dec-21	Yes	-	-	-	-
Postnatal care	NG194	20 April 2021	20-Apr-21	Yes	-	-	-	-
Pregnancy and complex social factors: a model for service provision for pregnant women with complex	CG110	22 September 201	22-Sep-10	Yes	-	-	-	-
Preterm labour and birth	NG25	20 November 2015	10-Jun-22	Yes	-	-	-	-
Prostate cancer: diagnosis and management	NG131	9 May 2019	15-Dec-21	Yes	-	-	-	-
Twin and triplet pregnancy	NG137	4 September 2019	09-Apr-24	Yes	-	-	-	-
Urinary incontinence and pelvic organ prolapse in women: management	NG123	2 April 2019	24-Jun-19	Yes	-	-	-	-
